# Prophylactic Effect of Lamivudine for Chemotherapy-Induced Hepatitis B Reactivation in Breast Cancer: A Meta-Analysis

**DOI:** 10.1371/journal.pone.0128673

**Published:** 2015-06-09

**Authors:** Wei Tang, Lun Chen, Ruohui Zheng, Lingxiao Pan, Jin Gao, Xigang Ye, Xiaoshen Zhang, Wenbo Zheng

**Affiliations:** 1 Department of Breast Surgery, The First Affiliated Hospital of Guangzhou Medical University, Guangzhou, 510120, People’s Republic of China; 2 Department of Biotechnology, School of Life Science, Sun Yat-sen University, Guangzhou, 510275, People’s Republic of China; Centers for Disease Control and Prevention, UNITED STATES

## Abstract

**Background:**

Three strategies using lamivudine have been proposed to prevent chemotherapy-induced HBV (hepatitis B virus) reactivation in the clinical setting. The purpose of this meta-analysis is to evaluate the efficacy of the early preemptive strategy, deferred preemptive strategy and therapeutic strategy in patients with HBsAg-positive breast cancer during chemotherapy.

**Methods:**

Clinical studies published from database inception until Nov 1, 2014, were included for analysis. The primary outcomes were overall survival, rate of chemotherapy disruption and virological and clinical reactivation. The secondary outcomes were the rates of HBV-related chemotherapy disruption, HBV-related mortality, YMDD mutations and withdrawal hepatitis.

**Results:**

Four hundred and thirty patients in four studies that compared the early preemptive strategy with a therapeutic strategy were included. Application of early preemptive lamivudine was superior in reducing HBV recurrence (pooled OR: 0.12, 95% CI, 0.04 to 0.31, P< 0.0001), the incidence of HBV-related hepatitis (pooled OR: 0.13, 95% CI, 0.04 to 0.37, P< 0.0001) and the rate of chemotherapy disruption (pooled OR: 0.37, 95% CI, 0.23 to 0.60, P< 0.0001). In these two groups, no significant difference was found in overall mortality (P = 0.32), YMDD mutant rate (P = 0.13) or incidence of withdrawal hepatitis (P = 0.38). Of the two studies that compared the efficacy of an early and a deferred preemptive strategy, one showed that an early preemptive strategy significantly reduced the incidence of hepatitis (P = 0.046), whereas the other showed no significant difference (P = 0.7).

**Conclusions:**

An early preemptive strategy is superior to a therapeutic strategy in decreasing the incidence of HBV reactivation, incidence of HBV-related hepatitis and rate of chemotherapy disruption in patients with breast cancer. A deferred preemptive strategy might be an alternative approach to controlling viral replication.

## Introduction

The reactivation of HBV(hepatitis B virus) replication in HBsAg-positive patients caused by chemotherapy has been observed for decades[1, 2]. The chemotherapy-induced liver damage is characterized by enhanced HBV replication and widespread infection of hepatocytes followed by rapid immune-mediated destruction. It is thought that viral replication precedes the clinical hepatitis flare-up by a few weeks[3]. Upon the reactivation of HBV, the clinical consequences range from asymptomatic hepatitis to fatal hepatic failure. It may cause not only HBV-related mortality or morbidity but also interruption or early termination of planned chemotherapy, which might compromise a patient’s prognosis.

**Fig 1:**
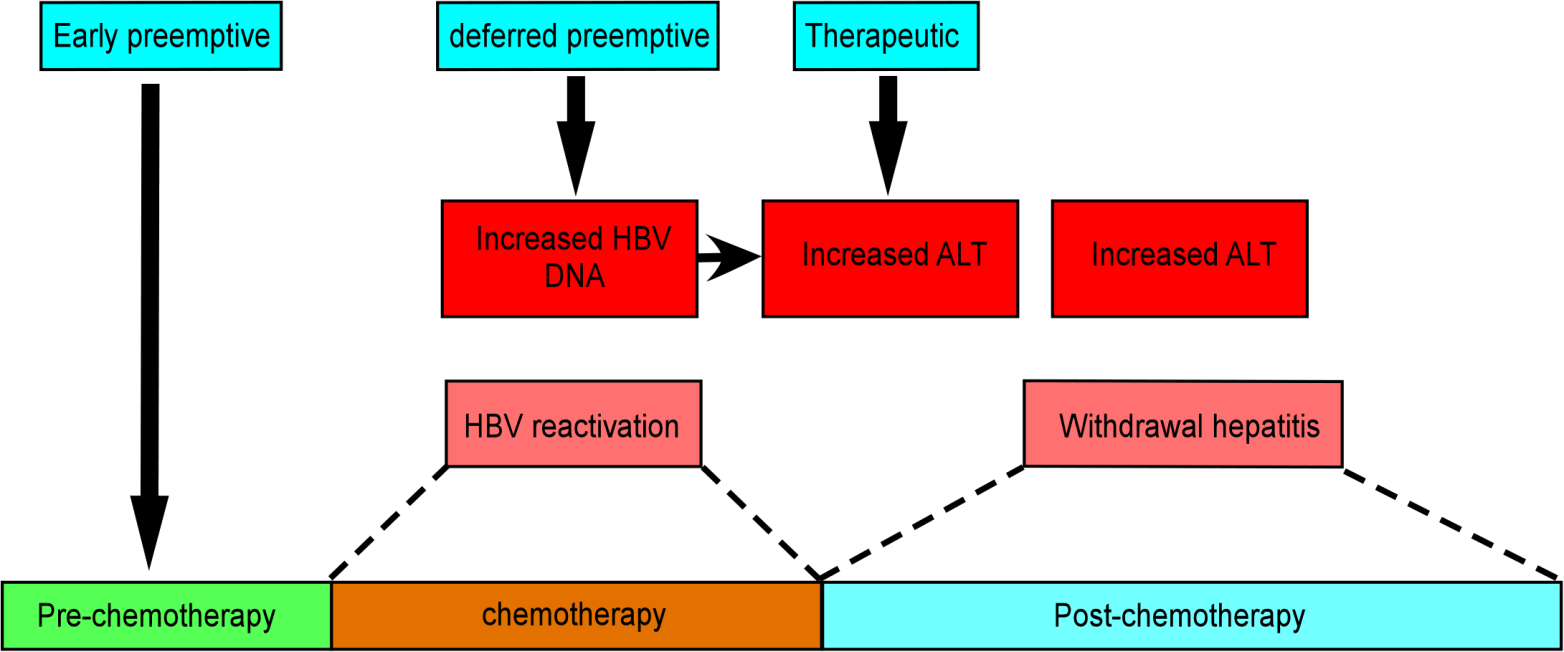
The proposed strategies to interfere with HBV during the chemotherapy in breast cancer patient. The phases of the patients had been divided into three stages; pre-chemotherapy, chemotherapy and post-chemotherapy. Lamivudine is given at the commencement of chemotherapy in early preemptive strategy and is given only after a significant rise of HBV DNA level in deferred preemptive strategy. If no early or deferred preemptive application of lamivudine had been given but the HBV DNA and ALT increased, the lamivudine applied to patients was used for therapeutic purpose.

As an effective antiviral agent, lamivudine has been used to treat patients who develop HBV reactivation during chemotherapy[4, 5]. This therapeutic strategy of starting lamivudine treatment after development of a clinical hepatitis flare-up has been reported to have a role in controlling HBV reactivation during chemotherapy[5, 6]. However, another report considered that this approach might result in fatal reactivation or a compromise in prognosis due to chemotherapy disruption[7]. Meanwhile, two other preemptive strategies have been employed to prevent chemotherapy-induced hepatitis: early preemptive strategy and deferred preemptive strategy(Fig 1). The early preemptive strategy, in which lamivudine is given at the commencement of chemotherapy, had been reported to prevent the recurrence of HBV-related hepatitis, with less HBV reactivation and chemotherapy disruption. In the deferred preemptive strategy, patients receive lamivudine only after a significant rise of HBV DNA level during chemotherapy. It was reported that this approach might have comparable efficacy in suppressing HBV reactivation and preventing a chemotherapy-induced hepatitis to the early preemptive strategy.

Some studies have been conducted to compare the efficacy of these three strategies in breast cancer patients who received chemotherapy, but the application of lamivudine for HBV reactivation remains controversial. To gain a deeper insight into this issue, we performed this meta-analysis to assess the efficacy of lamivudine prophylaxis in reducing hepatitis complications in HBsAg-positive patients with breast cancer undergoing chemotherapy. In addition, the emergence of tyrosine-methionine-aspartate-aspartate (YMDD) mutation and withdrawal hepatitis were examined in this analysis.

## Materials and Methods

### Study searching

All relevant articles were retrieved from PubMed, Embase, MEDLINE, Ovid and the Central Registry of Controlled Trials of the Cochrane Library using a combination of the terms "chemotherapy", "lamivudine", "cancer", "carcinoma", "neoplasm", "malignant" and "breast". Two authors (LC and JG) applied the inclusion criteria, searched the literature, and extracted the data independently without restriction of language or year.

### Inclusion and exclusion criteria

Studies were included that compared lamivudine as a prophylactic or preemptive treatment (as defined in each trial) for HBsAg-positive patients with breast cancer who received systemic chemotherapy.

Exclusion criteria included: (1) case reports, reviews and conference reports; (2) studies without a control group; (3) studies that were unable to provide clear baseline characteristics; (4) patients with HCV, HDV or HIV co-infection.

### Outcomes and definitions

The primary outcomes were: (1) the rate of HBV reactivation, which was defined as an increase in HBV DNA levels more than 10 times or an absolute increase of HBV DNA levels that exceeded 1×10^9^ copies/ml[8]; (2) incidence of hepatitis, which was defined as a greater than three times increase in alanine aminotransferase (ALT) that exceeded the upper limit of normal range (ULN) or an absolute increase of ALT of more than 100 U/L[8]; (3) rate of chemotherapy disruption, which was defined as either a premature termination of chemotherapy or a delay of more than 8 days of chemotherapy between cycles[8] and (4) overall mortality.

Secondary outcomes included incidence of HBV-related hepatitis, rate of HBV-related chemotherapy disruption, HBV-related mortality, occurrence of YMDD mutations and withdrawal hepatitis.

### Data extraction and quality assessment

Two independent investigators performed the data extraction after the eligible studies were identified. Data discrepancies between the two reviewers were resolved by a third investigator, and a consensus was achieved for all data prior to the meta-analysis. Methodological quality assessment of the RCTs included was assessed using the Jadad quality scale. For cohorts, the quality of studies was assessed by the Newcastle-Ottawa Scale (NOS) with the following indicators: selection of cohorts, comparability of cohorts and assessment of the outcomes[9].

### Statistical analysis

Study results were shown as odds ratio (OR) with 95% confidence intervals (CIs). Statistical heterogeneity was evaluated by the chi-square and I-square (I^2^) tests, with significance set at P< 0.10. A random-effects model was used to pool results throughout the review as studies with varying designs were pooled. Additionally, sensitivity analysis was carried out. Statistical significance was regarded as P< 0.05. The Cochrane Collaboration's Review Manager Software (version 5.0 for Windows; the Cochrane Collaboration, Oxford, UK) and Stata version 12 (Computer Resource Center, Atlanta, Ameriman) were used for data analysis.

## Results

### Description of studies

**Fig 2:**
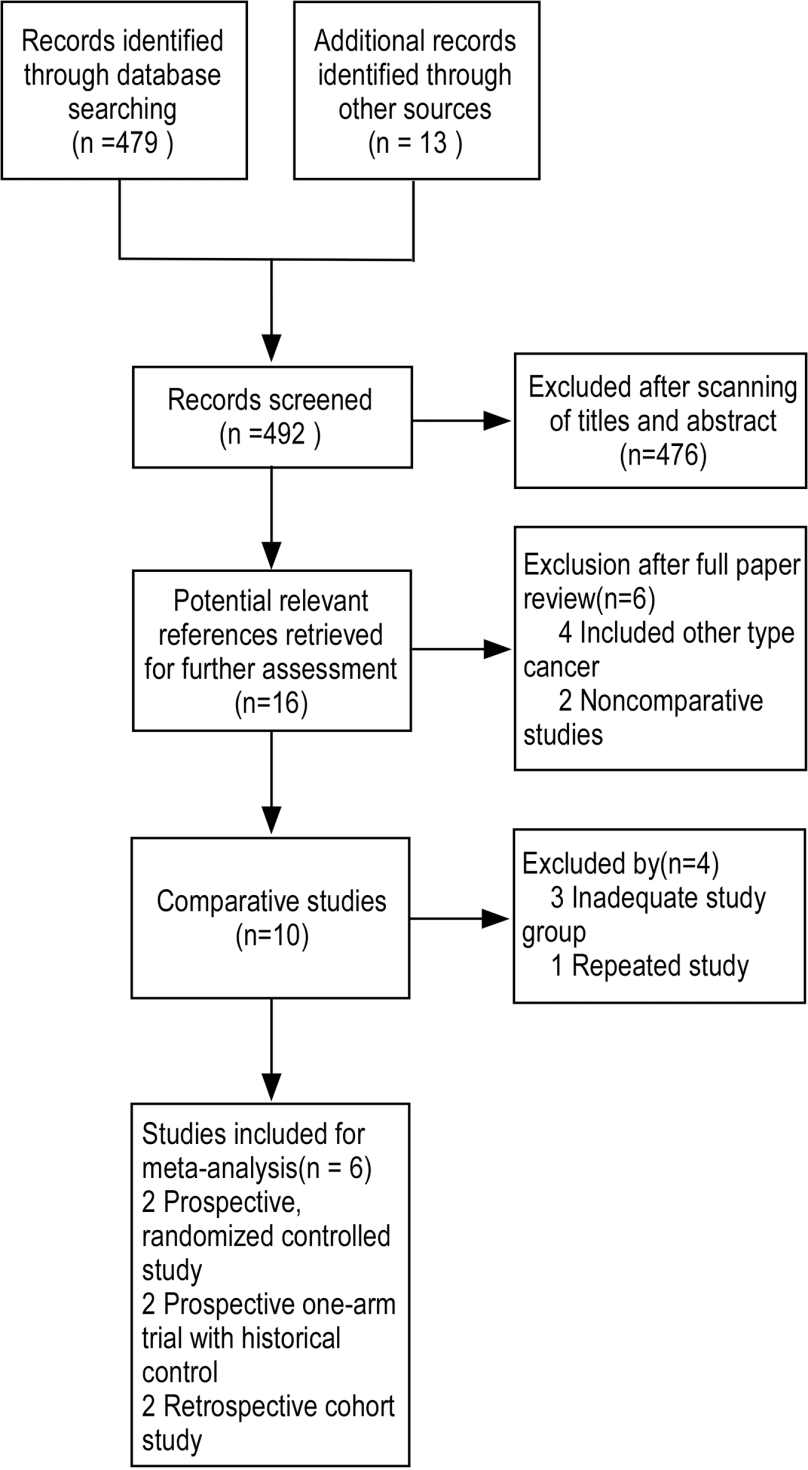
Schematic illustration of strategy to identified the subjects meeting the requirement in this study. Totally 492 records had been identified by literature searching. Based on abstracts, only 16 articles had been viewed as potential references for further assessment. After reviewing these articles in detail, only 6 articles had been selected for the meta-analysis.

A flow diagram of the literature search is summarized in Fig 2. Ultimately, four studies that compared early preemptive lamivudine administration with therapeutic lamivudine strategy were included in this study. These four studies included 1 prospective randomized controlled study[10], 2 retrospective cohort studies[11, 12] and 1 prospective one-arm trial with a historical control[13]. Another two studies that evaluated early preemptive administration of lamivudine compared with a deferred preemptive strategy were also included. One was a prospective randomized controlled study[14], and the other one was a prospective one-arm trial with a historical control[15].

Baseline characteristics of the trials are summarized in Table 1. All of the patients from the six studies were HBsAg-positive and were from East Asia, with four studies from China and two from Korea. There were no significant differences in age and baseline ALT level among the studies. Three trials had enrolled patients with advanced diseases and, hence, salvage chemotherapy was performed in their studies[10, 13, 15]. The results of the trials are shown in Table 2.

**Table 1:** The baseline characteristics of the studies.

	Yeo et al. (2004)		Long et al. (2011)		Yun et al. (2011)		Lee et al. (2012)		Tsai et al. (2011)		Dai et al. (2004)	
Type of strategy	EP	TP	EP	TP	EP	TP	EP	TP	EP	DP	EP	DP
No. of patients	31	61	21	21	55	76	73	92	23	22	11	9
Median age (range)	46(31–68)	46(31–71)	45(29–64)	43(20–62)	48(30–68)	46(30–69)	46(29–67)	45(29–72)	46.7	50.4	47(36–58)	43(27–55)
Median baseline ALT, (range), IU/l	28(13–137)	27(10–98)	22.3(7–96)	14.6(6–27)	25	25	20(6–50)	19(6–40)	NA	NA	14(12–31)	15(6–54)
Chemotherapy regimen												
Anthracycline based	30	36	2	1	28	45	71	61	NA	20	5	4
Taxane based	NA	NA	7	4	0	0	0	0	NA	0	0	2
Anthracycline and taxane based	NA	NA	10	16	27	31	NA	NA	NA	2	5	3
Others	NA	NA	2	0	0	0	NA	NA	NA	0	0	0
Use of glucocorticoids	23	36	NA	NA	28	33	47	43	23	22	NA	NA
Salvage chemotherapy included	Yes		Yes		No		No		No		Yes	
Type of trial	Prospective one-arm trial with historical control		Prospective, randomized controlled study		Retrospective cohort study		Retrospective cohort study		Prospective, randomized controlled study		Prospective one-arm trial with historical control	

ALT, alanine aminotransferase; EP, early preemptive group; TP, therapeutic group; DP, deferred preemptive group, NA, non available

**Table 2:** The results of the trials.

	Yeo et al.(2004)		Long et al. (2011)		Yun et al. (2011)		Lee et al. (2012)		Tsai et al. (2011)		Dai et al. (2004)	
Type of strategy	EP	TP	EP	TP	EP	TP	EP	TP	EP	DP	EP	DP
No. of patients	31	61	21	21	55	76	73	92	23	22	11	9
HBV reactivation	2	19	0	6	1	16	1	5	NA	15	0	5
Hepatitis	4	36	5	3	5	25	2	13	3	4	0	5
HBV-related hepatitis	2	19	0	0	1	16	1	5	NA	4	0	4
Chemotherapy disruption	5	28	4	2	2	11	36	66	0	2	NA	NA
HBV-related chemotherapydisruption	1	13	0	0	0	7	3	9	NA	NA	NA	NA
Delay of chemotherapy	5	18	3	1	2	9	36	56	NA	NA	NA	NA
Delay of chemotherapy due to HBV reactivation	1	18	2	1	0	5	3	7	NA	NA	NA	NA
Premature termination	1	10	1	1	0	2	0	10	NA	NA	NA	NA
Premature termination due to HBV reactivation	0	6	0	0	0	2	0	2	NA	NA	NA	NA
Lamivudine treatment course*	173.5 d	NA	NA	NA	185 d	NA	7.5 m	NA	27.5 w	16.1 w	6 m	NA
Lamivudine duration continued after chemotherapy completion*	8 w	8 w	8 w	8 w	2 m	NA	2.8 m	NA	4 w	4 w	4 w	4 w
Overall mortality	NA	NA	0	1	0	1	0	1	NA	NA	1	2
HBV-related mortality	NA	NA	0	0	0	0	0	1	NA	NA	0	1
YMDD mutations	NA	NA	0	0	2	0	1	0	NA	NA	0	0
Withdrawal hepatitis	0	0	0	0	1	0	0	0	1	0	0	0

EP, early preemptive group; TP, therapeutic group; DP, deferred preemptive group; NA, non available; d, days; w, weeks; m, months

*, mean

### Early preemptive strategy vs. therapeutic strategy

Four studies that compared an early preemptive lamivudine administration with a therapeutic lamivudine strategy were pooled (Table 3). A significant difference in HBV recurrence was observed between the two groups (pooled OR: 0.12, 95% CI, 0.04 to 0.31, P< 0.0001). Early preemptive lamivudine administration achieved a more favorable result for reducing the rate of HBV reactivation. In total, the rates of HBV reactivation were 2.2% in the early preemptive lamivudine group and 18.4% in the therapeutic lamivudine group. The P value was 0.79 for the heterogeneity, and the corresponding I^2^ statistic was 0%, which suggested low variability among these studies.

**Table 3:** Meta-analysis of the various outcomes.

	n	Patients		Chi^2^,P value;I^2^	Pooled OR, (95% CI),P value	Sensitivity Analysis
		EP(AN)	TP(AN)			Pooled OR, (95% CI), P value; Trials Omitted
HBV reactivation	4	180(4)	250(46)	1.04, 0.79; 0%	0.12, (0.04,0.3), < 0.0001	0.13,(0.04,0.37), 0.0001; Long et al.
Hepatitis	4	180(16)	250(77)	8.78, 0.03; 66%	0.23,(0.13, 0.41), < 0.00001	0.16,(0.08,0.31), <0.00001; Long et al.
HBV-related hepatitis	4	180(4)	250(40)	0.72, 0.70; 0%	0.13, (0.04,0.37), 0.0001	0.13,(0.04,0.37), 0.0001; Long et al.
Chemotherapy disruption	4	180(47)	250(107)	4.97, 0.17; 40%	0.37, (0.23,0.60), < 0.0001	0.31,(0.19,0.52), <0.00001; Long et al.
HBV-relatedchemotherapy disruption	4	180(4)	250(29)	1.52, 0.47; 0%	0.20, (0.07,0.57), 0.002	0.20,(0.07,0.57), 0.002; Long et al.
Delay of chemotherapy	4	180(46)	250(84)	3.17, 0.37; 5%	0.58, (0.36,0.95), 0.03	0.53,(0.32,0.87), 0.01; Long et al.
Delay of chemotherapy due to HBV reactivation	4	180(6)	250(31)	5.08, 0.17; 41%	0.28, (0.12,0.67), 0.004	0.20, (0.07,0.57), 0.002; Long et al.
Premature termination	4	180(2)	250(23)	2.24, 0.52; 0%	0.17, (0.05,0.56), 0.004	0.12,(0.03,0.52), 0.004; Long et al.
Premature termination due to HBV reactivation	4	180(0)	250(10)	0.12, 0.94; 0%	0.2, (0.03,1.10), 0.06	0.2,(0.03,1.10), 0.06; Long et al.
Overall mortality	3	149(0)	189(3)	0.02, 0.99; 0%	0.39, (0.06,2.53), 0.32	0.43,(0.04,4.22), 0.47; Long et al.
HBV-related mortality	3	149(0)	189(1)	Not applicable	0.41, (0.02,10.34), 0.59	0.41, (0.02,10.34), 0.59; Long et al.
YMDD mutation	3	149(3)	189(0)	0.08, 0.78; 0%	5.43, (0.60,48.95), 0.13	5.43,(0.60,48.95), 0.13; Long et al.
Withdrawal hepatitis	4	180(1)	250(0)	Not applicable	4.21, (0.17,105.33) 0.38	4.21,(0.17,105.33), 0.38; Long et al.

n, number of trials included; AN: actual number being compared; EP, early preemptive group; TP, therapeutic group

The incidence rates of hepatitis were 8.9% and 30.8% in the early preemptive lamivudine and therapeutic lamivudine groups, respectively (pooled OR: 0.23, 95% CI, 0.13 to 0.41, P< 0.0001). The P value was 0.03 for the heterogeneity, and the corresponding I^2^ statistic was 66%, which suggested low variability among these studies. The heterogeneity might be due to the trial of Long et al.[10], in which all cases of hepatitis were attributable to chemotherapeutic drugs. The meta-analysis showed that the therapeutic lamivudine group had a significantly higher rate of incidence of HBV-related hepatitis than the early preemptive lamivudine group (2.2% vs. 16%, pooled OR: 0.13, 95% CI, 0.04 to 0.37, P< 0.0001). The chi-square and I^2^ analyses showed no heterogeneity.

There were significant differences in chemotherapy disruption between the early preemptive lamivudine and the therapeutic lamivudine groups, including the rate of chemotherapy disruption (26.1% vs. 42.8%, respectively; pooled OR: 0.37, 95% CI, 0.23 to 0.60, P< 0.0001) and HBV-related chemotherapy disruption (25.6% vs. 33.6%, pooled OR: 0.20, 95% CI, 0.07 to 0.57, P = 0.002). It was suggested that the outcomes were in favor of the early preemptive lamivudine group. The results were similar in terms of the rate of premature termination of chemotherapy (PTC), HBV reactivation resulting from PTC, delay of chemotherapy (DC) and HBV reactivation resulting from DC. No significant heterogeneity was shown among studies. Moreover, no significant differences were observed between groups in terms of the overall mortality (P = 0.32) and HBV-related mortality (P = 0.59).

YMDD mutants were reported in 3 trials (Table 2). There was no statistically significant difference in the rate of YMDD mutation between the early preemptive lamivudine and the therapeutic lamivudine groups (2.0% vs. 0%, respectively; pooled OR: 5.43, 95% CI, 0.60 to 48.95, P = 0.13). With the early preemptive use of lamivudine, patients did not show higher rate of YMDD mutants compared with therapeutic therapy. Four trials provided data on the incidence of withdrawal hepatitis. The overall pooled estimate was not significant (pooled OR: 4.21, 95% CI, 0.17 to 105.33, P = 0.38)

### Early preemptive strategy vs. deferred preemptive strategy

Only two prospective studies compared the early preemptive use of lamivudine with a deferred preemptive strategy (Table 3). Dai et al.[15] analyzed the clinical outcome of 20 patients treated with either early preemptive use of lamivudine (11 patients) or deferred preemptive use of lamivudine (9 patients). No HBV reactivation or overt hepatitis occurred in the early preemptive group (0%) compared to the group with deferred preemptive lamivudine (55.6%) (P = 0.046). There was no significant difference between the two procedures in overall mortality (P = 0.476).

However, the incidence of hepatitis during chemotherapy (P = 0.7) and the rate of chemotherapy disruption (P = 0.23) were not significantly different between the two groups in Tsai's study[14]. Patients in the deferred preemptive group had a significantly shorter lamivudine treatment course during chemotherapy (P = 0.034). Therefore, although deferred preemptive strategy demonstrated less efficiency for prevention of HBV reactivation, we still believed this strategy could give patients some benefit such as reducing the complications or viral replication during HBV reactivation.

## Discussion

HBV reactivation during cytotoxic chemotherapy might cause varying degrees of hepatic impairment in patients with breast cancer. The prognosis of cancer could be compromised because of the disruption of chemotherapy. Therefore, several strategies using lamivudine have been proposed to address chemotherapy-induced HBV reactivation. This meta-analysis indicated that the preemptive use of lamivudine had a substantial efficacy in reducing HBV reactivation and hepatitis in patients with breast cancer undergoing chemotherapy. The therapeutic use of lamivudine could not change the patterns of HBV reactivation[7].

However, it should be noted that the rates of HBV reactivation and HBV-related hepatitis of breast cancer patients in the control group in our meta-analysis were 18.4% and 16%, respectively. In other words, approximately 85% of HBsAg-positive patients would not develop hepatitis during chemotherapy without lamivudine protection. Thus, a large proportion of patients with HBsAg-positive breast cancer might be over-treated. Additionally, increasing health care costs and exposing patients to the risk of lamivudine resistance developed due to prolonged treatment, with little additional benefit.

Whether all patients with HBsAg-positive breast cancer receiving chemotherapy must receive lamivudine prophylaxis is a matter of controversy. There is a need for a risk assessment to guide decisions about the preemptive use of lamivudine[16]. Several risk factors for HBV reactivation in patients receiving chemotherapy have been postulated, including virological factors (baseline HBV DNA level, HBV virological marker, presence of a precore mutant strain and viral genotype), host factors (gender, age, ALT level), and treatment factors (use of anthracycline and corticosteroids)[17,18]. A higher baseline HBV DNA level was postulated to predict subsequent HBV reactivation[18]. Dai has postulated that precore mutant HBV is prone to reactivation during or after chemotherapy[19]. In this study[15], all the patients who carried the precore HBV mutant strain in the control group developed HBV reactivation during chemotherapy. As previously reported, older patients might be more vulnerable to HBV reactivation than younger patients[20]. However, age is neither operable nor practical in helping to decide which patients should receive preemptive lamivudine in clinical practice. The use of anthracycline regimens or corticosteroids appeared to be another risk factor for HBV reactivation[21]. However, in Dai's and Sohn's studies, the use of anthracycline or dexamethasone did not seriously increase the rate of HBV reactivation[15, 22]. Taken together, no independent risk factor has been recognized to determine the necessity of the preemptive use of lamivudine prior to initiating chemotherapy in breast cancer patients.

As early prophylaxis requires a longer treatment course, the deferred strategy has been proposed. In the deferred preemptive strategy based on HBV DNA surveillance, patients receive lamivudine only after a significant rise in their HBV DNA level during chemotherapy. Although this strategy has preventive effects in HBV reactivations, its effect on overt hepatitis remains controversial, and no significant differences were found in the rate of chemotherapy disruption, overall mortality or YMDD mutants in counterpart groups in this study. In addition, the lamivudine treatment course would be shorter if the deferred preemptive strategy is adopted. It seems that deferred preemptive lamivudine based on serial monitoring of HBV DNA levels could be a comparable alternative to early preemptive strategy. However, the patients undergoing a deferred preemptive strategy might be at risk for prolonged use of lamivudine, which is required once a clinical hepatitis has developed. Moreover, Long et al. argued that the deferred preemptive strategy neglected patients with elevated baseline HBV viral levels and would impair the rate of HBeAg seroconversion in HBeAg seropositive patients[10]. These problems could be solved by selecting patients carefully during enrollment. Considering the above arguments, more clinical trials including bigger sample sizes should be conducted to evaluate the practical use of the deferred preemptive strategy.

The duration of lamivudine administration, which is related to the emergence of YMDD mutations and the occurrence of withdrawal hepatitis, is another concern with the use of lamivudine. The mean lamivudine treatment course was approximately 6 months in the early preemptive groups (Table 2). And there were no significant differences in the rate of YMDD mutants and the incidence of withdrawal hepatitis among the three groups. Each of the four patients with YMDD mutations were in early preemptive group and had received lamivudine for at least 18 months. This was a result of a prolonged lamivudine treatment course[23]. It has been reported that a YMDD mutation appeared in 12–20% of patients in the first year of treatment and that the resistance might rise to 32% after one year of treatment[24, 25]. Another concern is the occurrence of withdrawal hepatitis. Two patients in the early preemptive group developed hepatitis after lamivudine withdrawal in the studies of Tsai and Yun[11, 14]. The occurrence of withdrawal hepatitis might be related to the duration of lamivudine treatment and the development of YMDD mutation[26, 27]. Unfortunately, whether the two cases of withdrawal hepatitis were related to a lamivudine-resistant strain has not been investigated further by the authors.

The optimal duration of lamivudine treatment after the cessation of chemotherapy is another open issue. As level III evidence, the AASLD (American Association for the Study of Liver Diseases) has recommended that for HBV carriers receiving cancer chemotherapy or immunosuppressive therapy with baseline HBV DNA <2,000 IU/ml, antiviral therapy should be continued for 6 months after completion of chemotherapy or immunosuppressive therapy. Patients with a high baseline HBV DNA (>2,000IU/ml) level should continue antiviral treatment until they reach treatment endpoints, as in immunocompetent patients[28]. However, most of the studies in this meta-analysis did not follow these recommendations in clinical practice. Lamivudine was continued for 4 weeks in two studies[14, 15], 8 weeks in two studies[10, 13], a median of 2 (range 0–47) months in Yun's study[11] and a median of 2.8 (range 0–28.9) months in Lee's study[12] (Table 2). Premature withdrawal of lamivudine in our studies did not lead to higher rates of YMDD mutations or withdrawal hepatitis. However, seven patients in Dai’s and Lee’s studies showed an increase in HBV DNA after cessation of lamivudine, and they remained clinically stable without evident hepatitis. In addition, three of these patients had a brief rise in HBV DNA level for 3–3.5 months after cessation of chemotherapy. This notable finding suggested that serum HBV DNA levels should be monitored for at least 6 months after lamivudine withdrawal and that laboratory hepatic panel tests were not sufficient to prevent the occurrence of withdrawal hepatitis. Premature withdrawal of lamivudine might lead to a rapid rebound in viral replication, resulting in HBV-related mortality. Conversely, a prolonged course of lamivudine prophylaxis might lead to the development of resistant strains and could be an economic burden in developing countries.

The limitations of this study were that only two studies were prospective randomized trials. In addition, some advanced cases that received salvage chemotherapy were included in the studies, which resulted in some bias. Additionally, the studies did not include HBsAg-negative patients, and positive conversion of HBsAg could not be counted as HBV reactivation. These factors might have led to false negativity for HBV reactivation. Further studies should take the following issues into account: (1) identifying independent risk factors for HBV reactivation during chemotherapy; (2) determining the efficacy of deferred preemptive strategy; (3) determining the optimal duration of lamivudine treatment for preemptive antiviral therapy; (4) evaluating the preventive effects of other antiviral drugs such as adefovir dipivoxil, entecavir and telbivudine.

In conclusion, this meta-analysis demonstrated the superiority of the preemptive strategy of lamivudine use to the therapeutic strategy of lamivudine use in decreasing the incidence of HBV reactivation, incidence of HBV-related hepatitis and rate of chemotherapy disruption in patients with breast cancer undergoing chemotherapy. Premature withdrawal of lamivudine did not lead to higher rates of YMDD mutations or withdrawal hepatitis. A deferred preemptive strategy might be an alternative approach to controlling viral replication with the benefit of a shorter lamivudine treatment course.

## Supporting Information

S1 PRISMA Checklist (DOC)
